# The Positive Effect of Women’s Education on Fertility in Low-Fertility China

**DOI:** 10.1007/s10680-021-09603-2

**Published:** 2022-02-07

**Authors:** Shuang Chen

**Affiliations:** Department of Social Policy, London School of Economics and Political Science, Houghton Street, London, WC2A 2AE UK

**Keywords:** Women’s education, Fertility, China, Low fertility

## Abstract

Despite pervasive evidence of more educated women having lower fertility, it remains unclear whether education reduces women’s fertility. This study presents new evidence of the causal effect of women’s education on fertility from China, where fertility has remained below the replacement level since the early 1990s. To account for endogeneity, the study exploits the timing and varying intensity of China’s higher education expansion as exogenous sources of increase in women’s education. Using data from China General Social Survey (2010–2012), findings show that each year of women’s education induced by the higher education expansion increases the number of children ever born by 10%. According to the average marginal effects, each additional year of women’s education increases the number of children ever born by 0.14, decreases the probability of having no children by 3 percentage points, and increases the probability of having two or more children by 4 percentage points. Two mechanisms drive the positive effect of education: first, education does not cause an increase in the mean age at first marriage; second, among ever-married women, education increases their demand for children. Findings from this study have important implications for China and other low-fertility developing countries.

## Introduction

Extensive research has studied the relationship between women’s education and fertility in developing countries (Bongaarts, 2003; Cleland, 2002; Cleland & Rodriguez, 1988; Cochrane, 1979; Martin, 1995). Although this literature has presented pervasive evidence that more educated women have lower fertility, it remains unclear whether education reduces women’s fertility. The negative correlation between women’s education and fertility may be entirely due to unobserved family background factors that determine both education and fertility (Kramarz et al., 2021; Neiss et al., 2002; Nisén et al., 2013; Rodgers et al., 2008; Tropf & Mandemakers, 2017). More education may be a consequence (rather than cause) of lower fertility (Cohen et al., 2011; Marini, 1984; Stange, 2011). Due to unobserved heterogeneity and reverse causality, comparing the fertility of higher- and lower-educated women leads to a biased estimate of the effect of education on fertility, and taking it as causal evidence could yield misleading policy implications. Indeed, the few studies of the causal effect of women’s education on fertility, set in both developing and developed contexts, have generated mixed evidence. While some demonstrate a negative causal effect (Cygan-Rehm & Maeder, 2013; Fort et al., 2016; Kamhöfer & Westphal, 2019; Osili & Long, 2008; Sohn & Lee, 2019), others find that the negative effect disappears (Breierova & Duflo, 2004; Geruso & Royer, 2018; Kan & Lee, 2018; Kramarz et al., 2021; McCrary & Royer, 2011; Monstad et al., 2008) or even reverses to positive (Braakmann, 2011; Fort et al., 2016) as soon as endogeneity of women’s education is accounted for. This study presents new evidence of the causal effect of women’s education on fertility in a low-fertility developing context.

Since the early 1990s, the total fertility rate (TFR) has remained below the replacement level in China (Cai, 2010; Feeney & Yuan, 1994; Guo et al., 2019; Morgan et al., 2009; Zeng & Hesketh, 2016; Zhao & Zhang, 2018). Also during this period, women’s educational opportunities have increased rapidly (Hannum, 2005; Wu & Zhang, 2010; Yeung, 2013). Gender gaps in education have narrowed at the primary and secondary levels and reversed at the tertiary level (Hannum, 2005; Wu & Zhang, 2010; Yeung, 2013). In contrast to the rapid growth in educational opportunities, however, women’s disadvantages in the labour market have increased in terms of participation (Attané, 2012; Chi & Li, 2014; Hare, 2016), earnings (Chi & Li, 2014; Gustafsson & Li, 2000; Zhang et al., 2008), as well as occupational segregation (Chen et al., 2013; He & Wu, 2017; Zhang, 2013). Within the family, traditional gender ideologies and gendered division of labour remain unchanged (Ji et al., 2017; Ji, 2015a; Luo & Chui, 2018; Pimentel, 2016; Qian & Li, 2020; Zuo, 2003; Zuo & Bian, 2001), further exacerbating women’s disadvantages in the labour market and the work-family conflicts they face (Maurer-Fazio et al., 2007, 2011; Zhang et al., 2008; Zhang & Hannum, 2015).

Previous research on the relationship between women’s education and fertility in China is largely lacking, except for a few cross-sectional studies set in the 1980s or earlier (Lavely & Freedman, 1990; Zhang, 1990). The current study is set in a period (2010–2012) by which TFR had remained below the replacement level for almost two decades. Even though the period precedes the announcement of a universal two-child policy (Zeng & Hesketh, 2016), a large proportion of the population was already permitted to have more than one child (Gu et al., 2007; Zeng & Hesketh, 2016).^1^ Methodologically, this study exploits the timing and varying intensity of China’s higher education expansion in 1999 as a natural experiment to estimate the causal effect of women’s education on fertility.

## Theoretical Framework

According to Easterlin’s framework (Easterlin, 1975; Easterlin & Crimmins, 1985), fertility is determined by the potential supply of children, demand for children, and cost of fertility regulation. Easterlin’s original framework has been applied to examining the effect of women’s education on fertility in developing countries (Cochrane, 1979; Mason, 1987). Here, I consider the two most relevant pathways in the Chinese context where contraceptive use is prevalent among women (Zheng et al., 2012): age at first marriage and demand for children. Later in the article, I discuss whether more educated women are also better able to translate their demand for children into fertility regulation and actual fertility.

### Age at First Marriage

In theory, education delays women’s entry into marriage for three reasons. First, because school enrolment and motherhood are often incompatible, longer school enrolment has a mechanical effect of postponing family formation and childbearing (Bhrolcháin & Beaujouan, 2012; Black et al., 2008; Lappegård & Rønsen, 2005; Neels et al., 2017). Second, according to Becker’s economic theory of marriage (1973, 1991), more educated women have fewer gains from marriage, because higher earnings and labour market participation make the gendered division of labour within the household less advantageous. In many European countries and the USA, women’s education is not associated or even positively associated with marriage, countering Becker’s theory (Blossfeld & Kiernan, 2019; Blossfeld & Huinink, 1991; Isen & Stevenson, 2010; Sweeney, 2002). However, in countries with more traditional family systems and a high degree of role differentiation by gender, negative associations between women’s education and probabilities and rates of marriage continue to be found (Blossfeld & Kiernan, 2019; Ono, 2003). Across East Asian societies, marriage is laden with various traditional expectations of and for women, including childbirth, caretaking, and housework (Ji, 2015b). In this context, for more educated women, gains from marriage are fewer and the cost of marriage is higher. Moreover, preferences for women to marry men of higher status than themselves remain strong and the spouse-selection criterion remains gender asymmetrical. Consequently, improvement in women’s socioeconomic status relative to men’s results in marriage market mismatches for higher-status women and lower-status men (Raymo & Iwasawa, 2005; Raymo & Park, 2020; Yu & Xie, 2015). More educated women may also find it harder to find men who share their values (Jones, 2007), particularly since more educated Chinese women increasingly adopt gender-egalitarian ideologies while men’s values have been slower to change and have even become more traditional (Pimentel, 2016; Qian & Li, 2020). Taken together, education is hypothesized to reduce fertility by delaying women’s first marriage.^2^

Although many observational studies in China show that more educated women marry later (Ji & Yeung, 2014; Piotrowski et al., 2016; Tian, 2013; Yeung & Hu, 2016; Yu & Xie, 2015), education cannot be said to *cause* later marriage. If women have taken into account the negative impact of education on marriage prospects when making educational decisions, the positive relationship between education and age at first marriage may be spurious, even if the theoretical pathways between education and later marriage were true. For example, previous research in China suggests that parents who believe that girls’ wellbeing depends on a good marriage invest less in their daughters’ education (Li & Tsang, 2003), whereas some girls pursue more education as a conscious strategy to avoid early marriage (Seeberg, 2014). This means that higher and lower educated girls differ in unobserved ways that affect when they marry. In other words, more educated girls would have married later even without more education. Moreover, increased education could be a result of delayed entry into marriage or parenthood (Cohen et al., 2011; Field & Ambrus, 2008; Marini, 1984; Stange, 2011), and reverse causality would also have led to an overestimation of the delaying effect of education.

### Demand for Children

Education shapes women’s demand for children by affecting their income and the cost of children (Becker, 1965, 1969; Leibenstein, 1974; Willis, 1973), and the net effect is ambiguous in theory. By increasing women’s income, education allows women to afford more children. Besides women’s own wages, education may also increase income from other sources (for example, their husbands’ wages if more educated women marry more educated men). The income effect may be weakened if increased income leads parents to spend more on the “quality” of existing children rather than “quantity” (Becker, 1969; Becker & Lewis, 1973; Becker & Tomes, 1976).^3^ The income effect may also be offset by a negative substitution effect due to the increased cost of children. By increasing women’s wages, education increases the value of women’s time, and thus, the opportunity cost of raising children. In addition, as more educated women rely less on children for economic gains or old-age support (Mason, 1987), education decreases the value of children, further reducing the number of children women demand.

When women must choose between working and raising children, the negative substitution effect exceeds the income effect, and an inverse relationship between women’s education and the demanded number of children is most commonly observed in empirical studies (Schultz, 1997). However, this relationship changes if childrearing does not entirely rely on women’s time, for example, if women can purchase childcare services (Ermisch, 1989; Hazan & Zoabi, 2015). Outsourcing a part of childrearing to childcare services essentially alters the cost of children from the woman’s perspective (Ermisch, 1989; Hazan & Zoabi, 2015). In this case, even though education still increases the value of women’s time, it does not increase the cost of raising children as much (as if childrearing entirely relies on women’s time), leading to a less negative or even positive effect on fertility (Ermisch, 1989; Hazan & Zoabi, 2015).^4^ Consistent with the theoretical models, in Norway, thanks to increased access to heavily subsidized childcare facilities, the association between women’s education and completed fertility has become less negative over time (Kravdal & Rindfuss, 2008). In the USA where there is little or no government subsidies for childcare, Hazan and Zoabi (2015) demonstrate a *U*-shaped relationship whereby highly educated women, for whom the cost of childcare services relative to income is lower, have more children and work longer hours than women with intermediate levels of education. More broadly speaking, the negative effect of women’s education on fertility is moderated or reversed if women’s roles as workers and mothers are more compatible. Access to childcare is one way to reduce women’s role incompatibility (Du & Dong, 2013; Kravdal & Rindfuss, 2008; Richter et al., 1994; Rindfuss & Brewster, 1996; Wood et al., 2020). Labour market institutions also matter (Adserà, 2005, 2011; Ahn & Mira, 2002; Brinton & Oh, 2019). If education leads to women enjoying more gender-egalitarian couple arrangements and division of household labour, it can also contribute to a reversal in the education-fertility relationship from negative to positive (Anderson & Kohler, 2015; Esping-Andersen, 2017; Esping‐Andersen & Billari, 2015; Goldscheider et al., 2015).

## Endogeneity of Women’s Education

A major empirical challenge faced by research studying the effect of women’s education on fertility is that women’s education is endogenous. Higher- and lower-educated women may differ in unobserved ways that also affect their fertility (Amin & Behrman, 2014; Neiss et al., 2002; Nisén et al., 2013; Rodgers et al., 2008; Tropf & Mandemakers, 2017). Fertility and education decisions may be jointly made, and women who have or intend to have more children may discontinue education earlier (Cohen et al., 2011; Marini, 1984; Stange, 2011). In the Chinese context, the varying enforcement of family planning policies correlated with socioeconomic contexts (Cai, 2010; Gu et al., 2007) adds further challenges to estimating the causal effect of education.

One approach to studying the causal effect of education fertility is to compare identical twins who differ in education levels (Amin & Behrman, 2014; Kramarz et al., 2021; Nisén et al., 2013; Rodgers et al., 2008; Tropf & Mandemakers, 2017). Although the approach effectively controls for any unobserved family background factors, the effect of education on fertility may still be confounded by individual heterogeneity, that is, factors causing education to differ between twins may also cause the fertility differences. The within-twins approach may also be problematic in the Chinese context, as previous research found that couples misreport their children as twins to avoid the punishment for violating the one-child policy (Huang et al., 2016).

Another common approach exploits educational reforms as exogenous sources of change in women’s education and obtains an instrumental variable (IV) estimate of the effect of women’s education. Exploiting compulsory education law changes, studies have found a negative effect of women’s education on fertility in Germany (Cygan-Rehm & Maeder, 2013) and England (Fort et al., 2016), a null effect in Norway (Monstad et al., 2008), UK (Geruso & Royer, 2018) and Taiwan (Kan & Lee, 2018), and a positive effect across Continental Europe (Austria, Denmark, France, Italy, and the Netherlands) (Fort et al., 2016) and in the UK (Braakmann, 2011). Two studies using higher education expansion as an exogenous increase in probabilities of women having a college degree found a negative effect on fertility in Germany (Kamhöfer & Westphal, 2019) and South Korea (Sohn & Lee, 2019). In Indonesia, Breierova and Duflo exploit the timing and varying intensity of a school construction program as exogenous sources of change in women’s education and found no effect on the number of children ever born (Breierova & Duflo, 2004,2004). Using a similar identification strategy, Osili and Long (2008) found a negative effect of women’s education on the number of children born before age 25. Compared to the mixed evidence of the educational effects on completed fertility, there is more consistent evidence showing that women’s education reduces teenage births (Black et al., 2008; Cygan-Rehm & Maeder, 2013; Geruso & Royer, 2018; Grönqvist & Hall, 2013; Kırdar et al., 2018; Monstad et al., 2008). However, the negative effect on teenage births may not extend to later years (Geruso & Royer, 2018; Kırdar et al., 2018; Monstad et al., 2008).

In this study, I exploit the timing of China’s higher education expansion along with its regional variations in intensity as exogenous sources of change in women’s education. The strategy is analogous to the ones used by previous studies in Indonesia (Breierova & Duflo, 2004; Duflo, 2001, 2004), Nigeria (Osili & Long, 2008), Taiwan (Chou et al., 2010), Malawi and Uganda (Andriano & Monden, 2019) to study the causal effects of education on fertility, health, and labour market outcomes. The idea is that the expansion induces exogenous increases in women’s education across cohorts and that regions where the expansion is more intense should see more increases in women’s education.

## Data and Method

### Data

This study draws on the 2010, 2011, and 2012 waves of the China General Social Survey (CGSS). The CGSS is the first nationwide, comprehensive, large-scale social survey in China, launched jointly by Renmin University and the Hong Kong University of Science and Technology (Bian & Li, 2012). Because the Chinese government has introduced major changes to the “one-child” policy since the end of 2013, restricting the data to before 2013 ensures that the policy environment is relatively stable during the period of the current study. In other words, no period effect associated with the changing fertility policy confounds the educational effect. I restrict the sample to women born between 1971 and 1989 and who are aged at least 23 at the time of the survey. I matched the individuals in the survey with province-level data using the province of *hukou* registration. Table 1 presents the descriptive statistics of the variables used in the analysis. The average years of education attained by the women in the sample are about 10 years, with 26% having attained some tertiary education or above and 17% with a high school degree. These are broadly in line with education statistics from other data sources (OECD, 2020; UNESCO, 2020). “Appendix Table 6” provides additional descriptive statistics of the number of children ever born by educational levels.

**Table 1:** Descriptive statistics of variables used in the analysis. *Source*: provincial-level data from China Statistical Yearbooks; individual-level data from China General Social Survey (CGSS) 2010, 2011 and 2012

*Provincial level*			
	*N*	Mean	SD
Rate of higher education expansion	31	0.25	0.03
Higher education enrolment in 1998 (log)	31	11.28	1.03
GDP in 1998 (log)	31	7.50	

	*N*	%
Intensity of fertility policy		
< 1.3	6	19.35
1.3–1.5	12	38.71
1.5–2	7	22.58
above 2	6	19.35

*Individual level*			
	*N*	Mean	SD
Years of education	4566	10.27	4.07
Age at time of the survey	4566	32.47	5.31
Children ever born	4566	1.18	0.79
Age at first marriage among ever married	4013	22.86	2.98
Demand for children among ever married^a^	3244	1.79	0.64

	*N*	%
Level of education attainment		
Primary or below	1041	22.80
Middle school	1542	33.77
High school	785	17.19
Tertiary or above	1198	26.24
Cohorts (according to age in 1998)		
Treated: aged below 18	1711	37.47
Partially treated: aged 18–22	1204	26.37
Control: aged 23–27	1651	36.16
Hukou status		
Non-agricultural	1119	24.51
Agricultural or other	3447	75.49
Ethnicity		
Han majority	4138	90.63
Non-Han minority	428	9.37
Children ever born		
0	784	17.17
1	2415	52.89
2	1169	25.6
3 or more	198	4.34
Marital status		
Ever married	4056	88.87
Never married	508	11.13

^a^The demanded number of children was not collected in the CGSS 2011 survey

### Measurement

The main independent variable of interest is years of education, which is converted from the level of educational attainment reported by the respondent (0 years for no education or less than primary level, 6 years for primary school, 9 years for middle school, 12 years for high school including technical/vocational high school, 15 years for vocational/technical college, and 16 years for a 4-year college or postgraduate degree). The main outcome of interest is the number of children ever born. Two intervening variables are examined directly. One is the age at first marriage. The other is the demand for children among ever-married women, which is measured by the woman’s answer to the question: “In the absence of the one-child policy, how many children do you wish to have?” Previous research has shown that this measure strongly predicts subsequent fertility behaviour in the Chinese context (Hermalin & Liu, 1990; Jiang et al., 2015; Zheng, 2014).^5^ Because reported demand for children might be biased by the rationalization of the existing number of children, an additional measure of demand for children is whether the woman demands more than two children.

### Analytic Strategy

#### China’s Higher Education Expansion

**Fig. 1:**
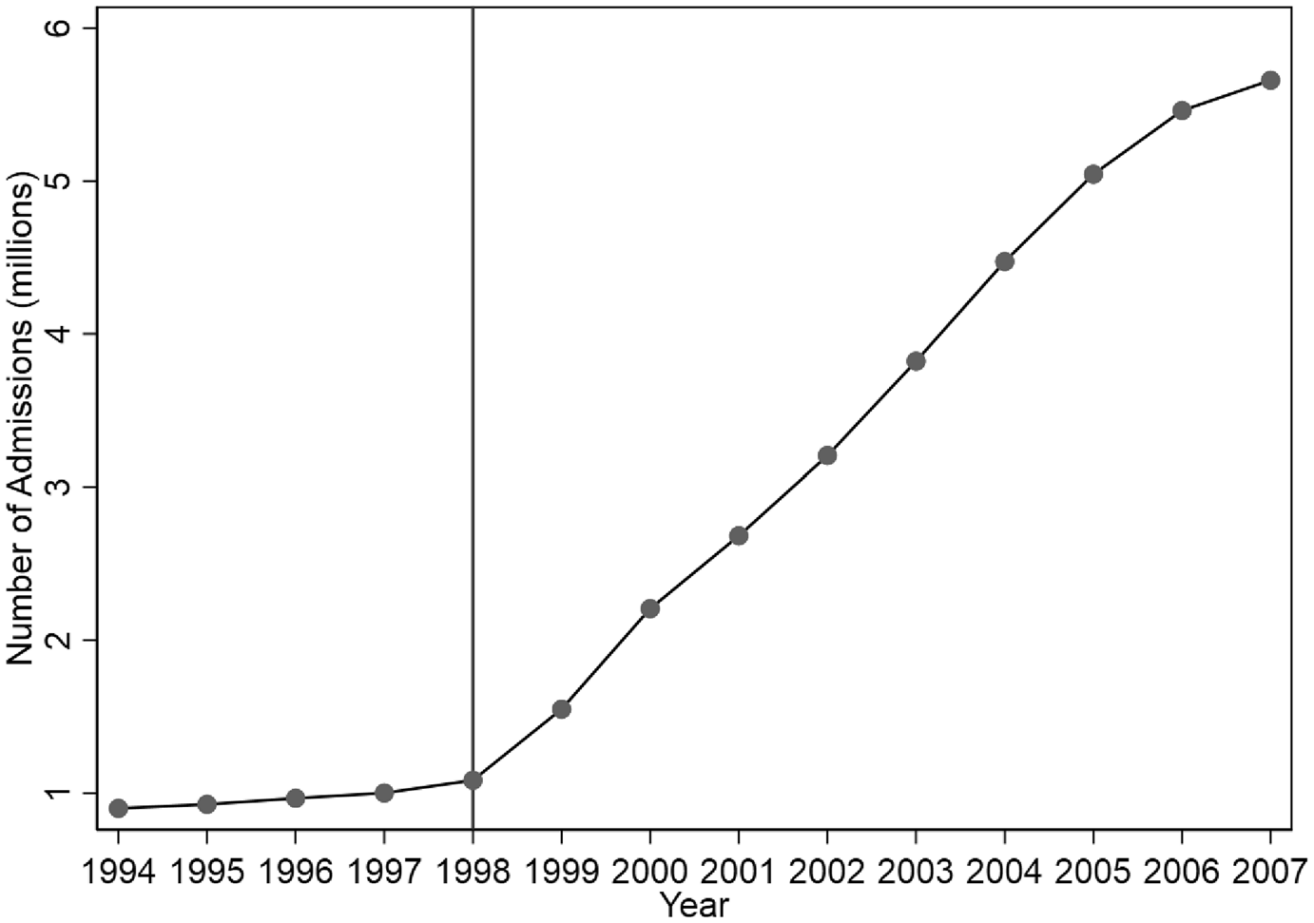
Number of admissions to higher education institutions nationwide by year. *Source*: China Statistical Yearbooks (1994–2007)

Students wishing to go on to higher education in China take the annual college entrance examination (also known as *Gaokao*) generally at the end of high school. Admission quotas for each province, university, and subject are negotiated annually between universities and national and provincial authorities. The cut-off scores for entry into each university and department are determined after all the test scores are known. In June 1999, right before the annual college entrance examination took place, the Ministry of Education made a sudden announcement to increase the number of admissions from 1.08 million in 1998 to 1.56 million in 1999, a 44% increase (Ministry of Education, 2004). The initial policy shock evolved into a sustained expansion until 2007. As illustrated in Fig. 1, between 1998 and 2007, the number of admissions to regular higher education institutions has seen more than a fivefold growth from 1.08 to 5.66 million. The higher education expansion has induced two sources of variation in women’s education: (1) the increase in the number of admissions over time and (2) the varying intensity of the expansion across provinces. In other words, provinces where the higher education expansion is more intense should see greater increases in women’s education across cohorts. The analytic strategy thus exploits the differential *growths* in women’s education across provinces induced by the higher education expansion to identify the effect of women’s education on fertility.

More specifically, given the modal age of taking the college entrance examination at 18, I define as “treated” cohort women who are aged below 18 in 1998. These individuals are most likely to benefit from the sudden increase in admissions to higher education. I define as “control” cohort women who are aged 23–27 in 1998. These individuals most likely had left school before the expansion started, and hence had missed the expansion. Due to the variation in school-starting ages and prevalent grade skipping and repetitions, the actual age at which a student takes the college entrance exam can vary. A minority of students may also decide to re-take the exam the following year if they are unsatisfied with the outcome of their first attempt. For example, over 30% of the applicants who sat the college entrance examination in 1997 had graduated from high school the year before or even earlier (Ministry of Education, 1998). Therefore, I define a “partially treated” cohort that contains women aged between 18 and 22 in 1998. Women’s education is expected to increase from the “control” cohort to the “partially treated” cohort and further to the “treated” cohort.

The intensity of the higher education expansion is measured by the annualized relative change in the number of admissions between 1998 and 2003, ranging from 16% in Beijing to 32% in Jiangxi province. In China, students are required to take the college entrance examination in the province of their *hukou* registration and subject to the admission quotas and the resulting cut-off scores specific to that province. The same requirements apply to migrant students even if they have been attending school in a different province.^6^ This institutional feature allows me to determine a woman’s exposure to the intensity of the expansion by the province of her *hukou* registration.^7^ Together, the interaction terms between the cohort indicators and the province-specific rate of expansion form the instrumental variables for women’s years of education.

Importantly, the expansion of higher education should not only increase attainment at the tertiary level but also the preceding levels of education. Research in China suggests that the perceived chance of admission to college affects decisions to drop out of the education system as early as the transition from middle school to high school (Loyalka et al., 2017; Yeung, 2013). Secondary level education in China mainly serves as a selection mechanism for tertiary education and prepares students for the college entrance exam (Li et al., 2012). Research outside China has also shown that an increased likelihood of getting into college increases students’ educational achievement and attainment at pre-collegiate levels (Akhtari et al., 2019; Khanna, 2019). Therefore, higher education expansion should not only benefit high school graduates but also incentivize students at lower levels of education to obtain more education.

#### Model Specifications

For the main outcome, children ever born, denoted $Y_{ijk}$, I estimate a Poisson model with multiplicative errors:

1 $$Y_{ijk} = \exp \left( {\alpha_{j} + \beta_{k} + \delta {\text{ED}}_{ijk} + x^{\prime}_{ijk} \gamma + \tau_{ijk} } \right)$$

where ${\text{ED}}_{ijk}$ denotes the years of education attained by individual $i$ with *hukou* registered in province $j$ and birth cohort $k$. $\alpha_{j}$ is province fixed effect, which controls for any time-invariant provincial characteristics (observed or unobserved).$\beta_{k}$ is a 1-year birth cohort fixed effect, which captures trends in women’s education not associated with the timing of the intensity of the higher education expansion. $x^{\prime}_{ijk}$ is a vector of covariates. Because the year of education, ${\text{ED}}_{ijk}$, is endogenous, I identify its effect using instrumental variables, specified as follows:

2 $${\text{ED}}_{{{\text{ijk}}}} = \alpha_{j} + \beta_{k} + z_{jk}^{^{\prime}} \lambda + x^{\prime}_{ijk} \gamma + \varepsilon_{ijk}$$

where $z_{jk}^{^{\prime}}$ is the instrumental variable, which is a vector of interaction terms between the cohort indicators (treated, partially treated) and the intensity of the expansion of province $j$, measured by the 5-year expansion rate in the number of admissions from 1998 to 2003.

For the cohort-intensity interactions to be a valid instrument for women’s education, a key assumption is that *trends* in women’s education would be the same in high-intensity and low-intensity provinces even in the absence of higher education expansion. There remain two potential threats to the “parallel trends” assumption. First, provinces with a lower baseline level of tertiary enrolment may see faster growth in women’s education even without the higher education expansion. Similarly, the rising demand for education may have been driven by economic growth rather than the higher education expansion, and more admission quotas may have been allocated to provinces where women’s education was increasing anyway. Second, the treated cohort of women was also exposed to the “one-child” policy introduced in 1979. Since declines in family sizes may also increase educational attainment due to the “quantity-quality trade-off” (Li et al., 2008), women’s education may have grown faster in some provinces than others due to the varying intensity of the one-child policy rather than that of the higher education expansion. Therefore, I include in the vector of covariates, $x^{\prime}_{ijk} \gamma$, the interactions between the cohort indicators and the number of tertiary enrolments in 1998, the provincial GDP in 1998, and the one-child policy intensity measure derived from Gu et al. (2007). By including these covariates, I further relax the identification assumption by letting the *trends* in women’s education differ across provinces. In addition, to increase the precision of the estimates, I include three individual-level covariates: woman’s age at the time of the survey (modelled by a series of dummy variables for each year of age), whether she has a non-agricultural *hukou,* and whether she is Han majority as opposed to an ethnic minority. Because the instrumental variables are on the cohort-province level, controlling for these individual-level characteristics should not affect the validity of the instrument.

To estimate the effect of education on intervening variables, I use the same first stage specified in Eq. 2 to regress years of education on the instrumental variables. For age at first marriage, I change the Poisson model specified in Eq. 1 to a parametric survival model, which assumes that the survival time follows a log-normal distribution. Because age at first marriage is reported in years and some individuals have not yet married at the time of the survey, the model is estimated using interval regression, which is a generalized Tobit model for censored data (Cameron & Trivedi 2009, p. 530; Conroy, 2005). Among ever-married women, to estimate the effect of education on the demand for children, I use the same Poisson model specified in Eq. 1 above. To estimate the effect of education on whether the individual demands more than two children, I replace the Poisson model in Eq. 1 with a Probit model.

## Results

### First-Stage: Effects of Higher Education Expansion on Women’s Education

For the higher education expansion to be a valid instrument, first, it must induce changes in women’s years of education. Specifically, provinces with higher intensity of expansion should see more increases in women’s years of education. Table 2 presents the estimated effects of the higher education expansion on women’s educational attainment using Eq. 2. The *F*-statistics test the null hypothesis that the coefficients on the interaction terms between cohorts and provincial rates of expansion are jointly zero. Model 1 is a baseline model containing only the province- and cohort-fixed effects, with no covariates. The direction and the magnitude of the coefficients are consistent with the identification assumption: provinces with more intense higher education expansion see greater increases in women’s years of education across the control, partially treated, and treated cohorts. Model 2 controls for individual-level covariates such as age at the time of the survey, *hukou* type, and ethnicity. Because the instrumental variables used in this analysis are on the cohort-province level, adding these individual-level covariates to the model should incur little change to the coefficients on the instrumental variables, but improve the precision of the estimates. This is indeed the case, as the coefficients in Model 2 are similar to those in Model 1, but the standard errors are smaller. Model 3 further relaxes the identification assumption by controlling for the interaction terms between the cohorts and the tertiary enrolment levels in 1998, provincial GDP in 1998, and the intensity of the “one-child” policy. This increases the magnitude of the coefficients on the instrumental variables.

**Table 2:** Effect of higher education expansion on women’s years of education: coefficients on the interactions between cohorts and provincial rate of expansion from linear regression models (base category is “Rate of expansion × control”)

	Years of education		
	(1)	(2)	(3)
Rate of expansion × cohorts			
Rate of expansion × partially treated	5.318	5.866†	9.382**
	(3.209)	(2.956)	(2.902)
Rate of expansion × treated	14.39***	15.63***	21.80***
	(3.223)	(2.889)	(3.908)
*F*-statistics	10.84***	15.80***	15.56***
Covariates			
Age at the time of the survey	No	Yes	Yes
Non-agricultural hukou	No	Yes	Yes
Han majority	No	Yes	Yes
Interaction between cohort and 1998 GDP	No	No	Yes
Interaction between cohort and 1998 enrolment	No	No	Yes
Interaction between cohort and fertility policy intensity	No	No	Yes
*N*	4566	4566	4566

Control cohorts are aged 23–27, partially treated cohorts are aged 18–22, and treated cohorts are aged below 18 in 1998, just before the start of the expansion. All model controls for 1-year birth cohort and province fixed effects. Standard errors are in parentheses and clustered by province

^†^*p* < 0.10, **p < 0.01, ***p < 0.001. The F-statistic tests the hypothesis that the coefficients on the interaction terms are jointly zero

**Fig. 2:**
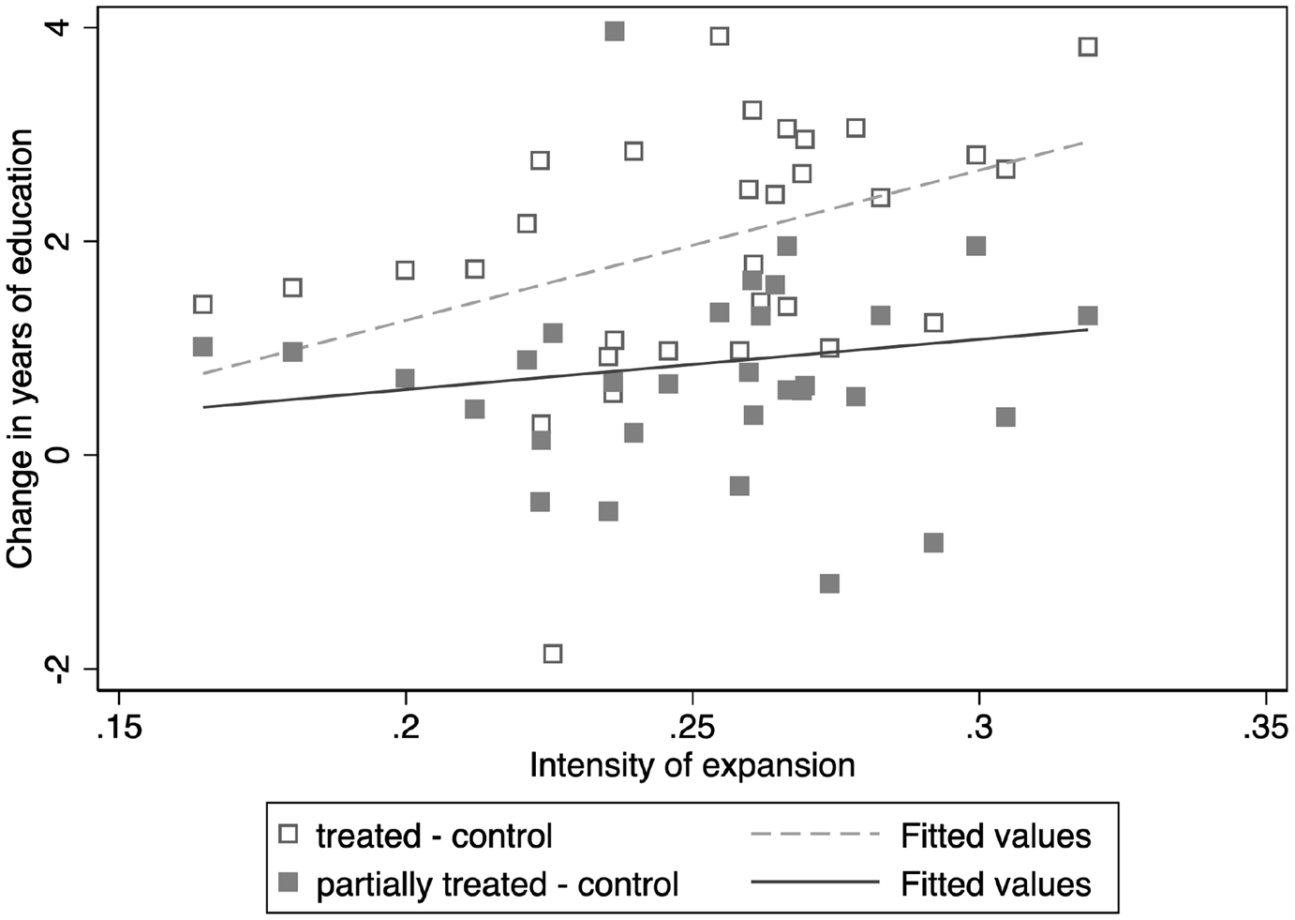
Scatterplot of intensity of higher education expansion versus change in years of women’s education (between control and partially treated cohorts, between control and treated cohorts) and two fitted lines using Eq. 2. *Source*: Author’s calculation using data from China Statistical Yearbooks (1994–2007) and CGSS (2010–12)

Across all three models, the *F*-statistic is greater than 10, indicating the strength of the instrument. According to Model 3, which is the full model used as the first stage in the following analyses, a province with a 10% point higher rate of expansion sees 0.9 additional years of increase in women’s education between the control and partially treated cohorts, and over two additional years of increase between the control and treated cohorts. Figure 2 visualizes the first-stage results by plotting the intensity of the higher education expansion versus the observed change in women’s years of education and overlaying the scatterplot with fitted lines using estimates from Model 3. Consistent with the identification assumption, the figure illustrates that provinces with higher rates of expansion have seen more increase (or less decrease) in women’s years of education across cohorts, and the slope of the fitted line for changes between the control and treated cohorts is steeper than that for changes between the control and partially treated cohorts. “Appendix Table 7” further illustrates that the higher education expansion not only increases women’s probability of completing tertiary education but also increases their probabilities of completing high school and middle school. Consistent with prior research in and outside China (Akhtari et al., 2019; Khanna, 2019; Loyalka et al., 2017), this means that increased admission to higher education not only benefits high school graduates but also increases the educational attainment of students at lower levels of education.

Another assumption required for the instrument to be valid is that the higher education expansion induces exogenous changes in women’s education. In the Appendix, I present sensitivity analyses that attest to the exogeneity assumption. The exogeneity assumption first implies that, in the absence of the higher education expansion, *trends* in women’s education should be the same between high-intensity and low-intensity provinces. I conduct a placebo test estimating the effect of the higher education expansion on the change in the education of women aged 28–32 in 1998 and those aged 23–27 in 1998. As expected, I find no effect on these cohorts of women who were exposed to the higher education expansion. The exogeneity assumption also implies that higher education expansion should not affect women’s fertility other than through increasing their years of education. In the sensitivity analysis presented in the Appendix, I discuss the potential threats. I present several direct and indirect tests, showing that the effect of women’s education found in this study is through increased years of education, not through changes in men’s education, quality of education, two-child policy eligibility, or any direct effect, lending support to the exclusion restriction assumption.

### Number of Children Ever Born

Table 3 presents results from a Poisson model and an IV-Poisson model estimated by a generalized method of moments estimators (Cameron & Trivedi, 2013; Mullahy, 1997; Windmeijer & Santos Silva, 1997). According to the Poisson model, women’s education is negatively associated with the number of children ever born. As soon as the endogeneity of education is accounted for in the IV-Poisson model, the coefficient flips from negative to positive: an additional year of education increases the number of children ever born by 10%. The Hausman test for endogeneity is statistically significant, meaning that education is indeed endogenous and that the Poisson model is inconsistent. Using the estimate from the IV-Poisson model, I calculate the average marginal effects of education: each year of education induced by the higher education expansion increases women’s number of children ever born by 0.14, decreases the probability of having no children by 3 percentage points, and increases the probability of having two or more children by 4 percentage points.

**Table 3:** Estimated effect of women’s education on children ever born

	Children ever born	
	(1)	(2)
	Poisson	IV-Poisson
Years of education	− 0.0436***	0.0982*
	(0.00404)	(0.0449)
Hausman test		10.05**
Overidentification test		0.861
*N*	4566	4566

Standard errors are in parentheses

All models control for 1-year birth cohort and province fixed effects, age at the time of the survey, non-agricultural hukou, interactions between cohorts and provincial level GDP in 1998, tertiary enrolment in 1998, and fertility policy intensity

*p < 0.05, **p < 0.01, ***p < 0.001

### Mechanisms

#### Age at First marriage

Table 4 presents the estimated effect of education on the logarithm of age at first marriage. According to the interval regression model (Column 1 Table 4), an additional year of education is associated with 1.4% older age at first marriage. However, the positive association disappears when an extended interval regression model with endogenous covariates is estimated (Column 2 Table 4). The correlation between errors from the structural equation (Eq. 1) and the first-stage equation (Eq. 2) is positive and significantly different from zero, meaning that education is endogenous.

**Table 4:** Estimated effect of women’s education on age at first marriage

	Age at first marriage (log)	
	(1)	(2)
	Interval regression	Interval regression with IV
Years of education	0.0140***	− 0.00912
	(0.000605)	(0.00766)
Correlation between errors		0.505***
*N*	4525	4525

Standard errors are in parentheses and clustered by province

All models control for 1-year birth cohort and province fixed effects, age at the time of the survey, non-agricultural hukou, interactions between cohorts and provincial level GDP in 1998, tertiary enrolment in 1998, and fertility policy intensity

***p < 0.001

#### Demand for Children

Table 5 presents models estimating the effect of education on the number of children demanded among ever-married women. The Poisson model indicates a slight negative effect of education on demand for children. However, in the IV Poisson model, the educational effect is positive, albeit not statistically significant. It is possible that responses to the demanded number of children are biased by the rationalization of children already had or the varying eligibility to have one or two children (Hermalin & Liu, 1990; Zhenzhen et al., 2009), which could have attenuated the estimated effect. To account for this possibility, the bottom panel of Table 5 presents the estimated effect on whether the individual demands more than two children. Consistently, while in the Probit model, an additional year of education is associated with a lower probability of demanding more than two children, the direction of the coefficient flips in the IV-Probit model: higher educational attainment significantly increases the probability of demanding more than two children. Both results suggest that, among ever-married women, education increases their demand for children.

**Table 5:** Estimated effect of women’s education on demand for children and whether demanding more than two children

	Demanded number of children	
	(1)	(2)
	Poisson	IV-Poisson
Years of education	− 0.00852*	0.0106
	(0.00403)	(0.0216)
Hausman test		0.813
Overidentification test		1.15
*N*	3244	3244

	Demand more than two children	
	(1)	(2)
	Probit	IV-Probit
Years of education	− 0.0429***	0.201**
	(0.0139)	(0.0632)
Wald test of exogeneity		5.07*
*N*	3095	3095

Standard errors are in parentheses and clustered by province

All models control for 1-year birth cohort and province fixed effects, age at the time of the survey, non-agricultural hukou, interactions between cohorts and provincial level GDP in 1998, tertiary enrolment in 1998, and fertility policy intensity

*p < 0.05, **p < 0.01, ***p < 0.001

## Discussion

### Interpreting the Positive Causal Effect of Education

Women’s education is negatively associated with the number of children ever born, however, as soon as endogeneity of education is accounted for, I find a positive causal effect of women’s education: each additional year of women’s education induced by the higher education expansion increases women’s number of children ever born by 10%. According to the average marginal effects, each year of women’s education increases the number of children ever born by 0.14, decreases the probability of having no children by 3 percentage points, and increases the probability of having two or more children by 4 percentage points. These results are consistent with those of Fort and colleagues (2016): their findings from Continental Europe demonstrate that each additional year of women’s education induced by the compulsory education reforms increases the number of biological children by 0.2–0.3 and decreases childlessness by 5–11% points. That the negative correlation between women’s education and the number of children ever born flips to positive in the IV-Poisson model is consistent with the expected direction of bias: if women who want to have more children also obtain less education, simply comparing the fertility of women with different education attainment overestimates the negative effect of education.

It is important to note that the IV estimator estimates the local average treatment effect (Angrist, 2001; Angrist et al., 1996; Imbens, 2014; Imbens & Angrist, 1994), which is the effect of education on “compliers”, or women who obtain more education because of the higher education expansion. While the local average treatment effect cannot be extrapolated to the overall population, for policymakers, it is more relevant than the average effect in the population, because the “compliers” are a subpopulation who are impacted by the policy reform (J. D. Angrist & Pischke, 2009; Card, 2001). Supply-side educational reforms tend to affect the schooling of individuals with relatively high returns to education (Card, 2001). Specifically, compliers of China’s higher education expansion have been shown to come from the more developed Eastern region, urban areas, and less likely to be ethnic minorities (Li & Xing, 2010). In this view, the positive effect of education found in this study echoes findings from the US that college completion increases fertility among the more advantaged women (Brand & Davis, 2011).

### Education Does Not Increase Mean Age at First Marriage

Why does women’s education increase the number of children ever born? The first mechanism revealed by the analyses is that women’s education does not increase the mean age at first marriage. Even though the study has found a strong correlation between women’s education and later age at marriage, the effect disappears as soon as the endogeneity of education is accounted for. This is consistent with the expectation that the correlation between more education and later age at marriage is drive by selection and reverse causality. Several studies set outside China have also shown that the relationship between education and postponement of marriage or first birth is not causal (Cygan-Rehm & Maeder, 2013; Fort et al., 2016; Kan & Lee, 2018; Lefgren & McIntyre, 2006; Neiss et al., 2002; Rodgers et al., 2008; Tropf & Mandemakers, 2017). Specifically, studies using within-identical twin models and set in the US, Denmark, and the UK illustrate that the association between education and later age at first birth is largely, if not completely, due to the unobserved family background factors that are related to both education and entry into parenthood (Neiss et al., 2002; Rodgers et al., 2008; Tropf & Mandemakers, 2017).

**Fig. 3:**
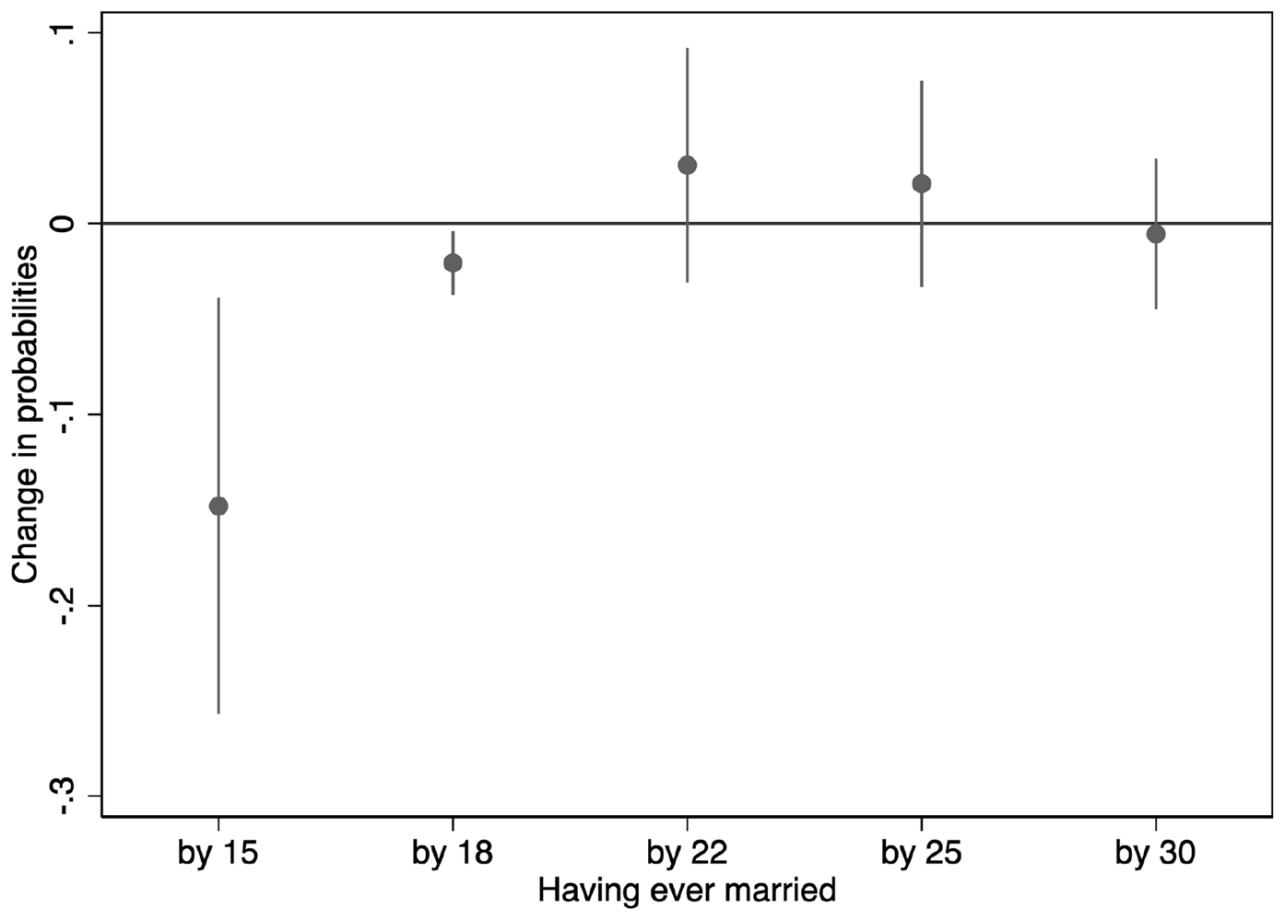
Average marginal effects of women’s education on probabilities of having entered first marriage by age 15, 18, 22, 25, and 30, estimated from IV-Probit models. *Source*: Author’s calculation

To further elucidate the null effect of women’s education on mean age at first marriage, I use IV-Probit models to estimate the causal effects of women’s education on the probability of having entered first marriage by age 15, 18, 22, 25, and 30, respectively. Figure 3 plots the average marginal effects calculated using estimates from these IV-Probit models. According to Fig. 3, women’s education significantly reduces the probabilities of having ever married by age 15 and 18, and yet, has a slightly positive, albeit non-significant, effect on probabilities of having ever married by age 22 and 25. By age 30, education makes no difference in probabilities of having ever married. This result suggests that the lengthening of school years does indeed delay marriage for women who would otherwise get married before age 18, consistent with theoretical predictions (Bhrolcháin & Beaujouan, 2012; Black et al., 2008; Lappegård & Rønsen, 2005; Neels et al., 2017). At the same time, however, Fig. 3 indicates a “catch-up” effect whereby women who postpone their marriage due to longer school enrolment get married as soon as the additional years of schooling are finished. As a result, despite reducing the probability of early marriage, women’s education does not cause an overall increase in the mean age at first marriage. Outside China, research has similarly found that even though education reduces early marriage or births, the negative effect may not extend to later years (Anderberg & Zhu, 2014; Fort, 2012; Geruso & Royer, 2018; Kırdar et al., 2018; Monstad et al., 2008).

### Education Increases Demand for Children

The analyses also reveal that education increases the demand for children among married women. As discussed earlier, women’s education can have a positive effect on demand for children if women’s roles as mother and worker are not incompatible. For example, with access to purchased childcare, more educated women not only have more children but also work more (Ermisch, 1989; Hazan & Zoabi, 2015; Rindfuss & Brewster, 1996; Wood et al., 2020). In “Appendix Table 8”, I show that education indeed increases ever-married women’s probability of being employed, and among those employed, each additional year of education increases their hourly wages by 15% according to the OLS model and 23% according to the 2SLS model. In other words, more educated women not only demand more children but also participate in the labour market more and earn higher wages. This is consistent with previous research from China, showing that higher-income women who can afford private childcare are better able to combine work and childrearing, while lower-income women are disproportionately disadvantaged by the work-family conflict (Ding et al., 2009; Du & Dong, 2013; Ji et al., 2017).

Education can also increase women’s demand for children by increasing their non-wage income. Compared to women’s own wages, the effect of non-wage income is less confounded by the opportunity cost of childrearing (Schultz, 1997). Particularly, if more educated women marry more educated men, as is the case in China (Han, 2010; Qian & Qian, 2014), education increases husbands’ wages. The top panel of “Appendix Table 9” estimates the effect of education on women’s education on non-wage income, that is, total household income minus women’s wage income. This includes but is not limited to husbands’ wage income. Education increases the woman’s non-wage income by 7.5%, and the estimate is even greater in the two-stage least squares (2SLS) model than in the OLS model. Moreover, using the husband’s education as a proxy for his earning potential, the bottom panel of “Appendix Table 9” further reveals that each year of women’s education increases the spouse’s education by 0.57 years.

If the higher education expansion and a sudden increase in the college-educated entrants into the labour market drives down the wage premium on tertiary education and increases unemployment rates (Knight et al., 2017), education may have increased the demand for children by *reducing* women’s labour market participation and wages. That education increases women’s employment and hourly wages as shown before (“Appendix Table 8”) rules out this explanation. It also means that the increased non-wage income has not led women to withdraw from the labour market or switch to lower-paying jobs.

Another alternative explanation is that the measure of demand for children is not reliable in China with policy restrictions on family size, which may have biased the estimated effect of education. For example, more educated women may be less subject to social desirability bias when interviewed about their demand for children, and thus less likely to underreport their true demand. To rule out this possibility, I examine responses to a different survey question which is likely subject to the same response bias. In the 2010 CGSS survey, respondents were asked how much they agree with the statement “the number of children one has is a personal matter. The government should not intervene.” If more educated women were less subject to response bias, they would be more likely to agree with the statement. Results from a multinomial logit model (“Appendix Table 10”) show that more educated women are no more or less likely than less educated women to agree or disagree with the statement. Therefore, there is no indication that the positive educational effect on the demand for children is due to response bias.

Taken together, the additional analyses presented here reveal that education not only increases women’s demand for children but also their labour market participation and wages. This suggests that more education may have enabled women to better combine work and childrearing. Education has also increased women’s non-wage income, including their husband’s wages through assortative mating, which may also help explain the positive effect of education on demand for children.

### Education Increases Women’s Resources Relative to Their Husbands'

If women had no say in the fertility decision-making process, their demand for more children would be irrelevant for their actual fertility. Previous research in China indicates that women with more education (Hu & Yeung, 2019) or income (Qian & Jin, 2018; Shu et al., 2013) relative to their husbands enjoy more power in making decisions regarding fertility and childrearing. Here, I investigate if education increases women’s resources relative to their husbands.’ “Appendix Table 11” illustrates that more educated women enjoy more resources relative to their husbands, measured by both education and income: education increases the probability of the woman having equal or more education than her husband, as well as the ratio of the woman’s income to her husband’s. To the extent that relative resources determine a woman’s fertility decision-making power (Hu & Yeung, 2019; Qian & Jin, 2018; Shu et al., 2013), this result suggests that education may have allowed women to better achieve their demand for children.

Of course, relative resources are not the only determinant of women’s decision-making power (Bittman et al., 2003; Cheng, 2019; Ferree, 1990; Shu et al., 2013). Whether a woman can translate her demand for children into contraceptive behaviours and actual fertility also depends on her husbands’ demand for children, disagreement between her and her husband’s demands, and communications between the couple (Bankole & Singh, 1998; Iacovou & Tavares, 2011; Mason & Smith, 2000; Riederer et al., 2019; Testa, 2012; Testa et al., 2014). Due to the lack of data, the current study is unable to investigate the effect of women’s education on fertility decision-making and regulation further.

## Conclusions

This study estimates the causal effect of women’s education on the number of children ever born in low-fertility China. Exploiting the timing and varying intensity of China’s higher education expansion, the study shows that each additional year of women’s education increases women’s number of children ever born by 10%. According to the average marginal effects, each additional year of women’s education induced by the expansion increases the number of children ever born by 0.14, decreases the probability of having no children by 3 percentage points, and increases the probability of having two or more children by 4 percentage points. The study further reveals two key mechanisms underlying the positive effect of women’s education: education does not increase women’s mean age at first marriage; among ever-married women, education increases their demand for children.

It is important to note that the outcome examined in this study is the number of children ever born, not completed cohort fertility. Given that the more educated women are also younger at the time of the survey, the effect on completed cohort fertility might be even more positive. Moreover, the effect estimated here is a local average treatment effect (Angrist, 2001; Angrist et al., 1996; Imbens, 2014; Imbens & Angrist, 1994) on women who obtain more education because of the higher education expansion, which cannot be extrapolated to the overall population. In addition, although the current study provides some suggestive evidence that education allows women to better achieve their demand for children, future research should investigate more directly whether education increases women’s fertility decision-making power, the role of their husbands, and the extent to which women’s demand for children translates into contraceptive behaviours and realized fertility.

Findings from this study have important policy implications for China. First, the positive causal effect of women’s education means that China’s fertility would have been even lower without the major progress in women’s education over the past decades. Previous research shows that the low period fertility in China has been driven by declines in first births (due to either delay in first births or increases in childlessness) offsetting the increases in second births (Guo et al., 2019; Zhao & Zhang, 2018). This study estimates that each additional year of women’s education induced by the higher education expansion has increased the probability of having at least one child by 3 percentage points and the probability of having two or more children by 4 percentage points. Second, the positive causal effect of women’s education implies that the current low fertility may not be irreversible. More specifically, education is shown to have increased women’s demand for children, not only by raising their income but also by reducing the work-family conflict they face. This echoes previous studies from China citing the high cost of childrearing as the key explanation for low fertility preferences, including the direct cost of education, lack of childcare provision, and the negative impact on women’s career (Basten & Jiang, 2015; Merli & Morgan, 2011; Zeng & Hesketh, 2016; Zhenzhen et al., 2009). Finally, findings from this study help illustrate why the transition from the “one-child” policy to a universal two-child policy has achieved a limited impact on raising fertility (Attané, 2016; Basten & Jiang, 2015; Guo et al., 2019; Zhao, 2015). While low fertility can be reversed, merely *allowing* women to have more children is insufficient without increased support for families or measures to reduce the work-family conflict women face.

Is the positive effect of women’s education on fertility unique to low-fertility China? A positive causal effect of women’s education on fertility has previously been found in Continental Europe (Fort et al., 2016) and the UK (Braakmann, 2011). While causal studies are few, cross-sectional studies from low-fertility Europe have found countries and subregions where more educated women have higher cohort fertility (Nisén et al., 2021), higher rates of second or higher-order births (Hoem & Hoem, 1989; Klesment et al., 2014; Kravdal, 1992; Wood et al., 2014, 2020), and higher fertility intentions (Testa, 2014). The negative correlation between women’s employment and fertility has also reversed in recent cohorts (Adserà, 2005; Ahn & Mira, 2002). It remains to be seen whether the positive causal effect of education on fertility found in China generalizes to other developing contexts, but the few existing studies suggest that the compatibility between work and childrearing has become a key determinant of women’s fertility in other low-fertility developing countries, too. For example, in Brazil, gender equality and the ability to combine work and childrearing are found to be positively associated with higher-order births (Castanheira & Kohler, 2017). In urban Thailand, the availability of alternative childcare arrangements for the firstborn has been shown to predict the probability of having a second child (Richter et al., 1994), and university-educated women now have the highest level of fertility intentions (Buathong et al., 2017). As more developing countries are expected to reach below-replacement fertility levels in the near future (United Nations et al., 2019), there is a compelling need for future research to better understand the effect of women’s education on fertility and help address the policy challenges posed by low fertility in the developing world.

## Appendix

### Sensitivity Analysis

#### Placebo Test

As discussed before, a key assumption for the instrumental variables to be valid is that, in the absence of the higher education expansion, *trends* in women’s education would not differ between high-intensity and low-intensity provinces. To further ensure that the differential growths in women’s education across provinces in the first-stage results are due to the higher education expansion rather than unobserved province-specific time-varying factors, I conduct a placebo experiment: I estimate the effect of the higher education expansion on the change in women’s education between the cohort aged 28–32 in 1998 and the cohort aged 23–27 in 1998. The expansion should have no effects on these cohorts of women because they had completed education before the expansion started. “Appendix Table 12” fits the same first-stage models on these two cohorts. A significant coefficient on the interaction term between cohort indicator and intensity of the expansion would imply either reverse causality (i.e. pre-existing trends in women’s educational attainment determines the province-specific intensity of higher education expansion) or that there are unobserved province-specific time-varying factors that have confounded the relationship between the higher education expansion and the trends in women’s education. “Appendix Table 12” shows that the estimated coefficient is not statistically significant in either of the three models, meaning that without the higher education expansion, the trends in women’s education between cohorts do not differ systematically between the high- and low-intensity provinces.

#### Exclusion Restriction

The exogeneity of the instrument also implies exclusion restriction, that is, higher education expansion does not affect women’s fertility other than through increasing their educational attainment. There are three main threats to this assumption. First, if higher education expansion affects men’s educational attainment too, it may affect women’s fertility outcomes not only through increasing their education but also through increasing the education of men. Second, higher education expansion may affect the quality of education women receive, and in turn, their fertility outcomes. Finally, the instrumental variables could have a direct influence on women’s fertility: for example, if provinces with more intense higher education expansion also have more intense enforcement of the one-child policy, then women born in these provinces are more likely to be only children and marry men who are only children. Because couples in which both partners are only-children are permitted to have two children (Zeng & Hesketh, 2016), these women may have more children not because they have obtained more education, but because they are more likely to be eligible to have two children.

“Appendix Table 13” presents direct evidence of the effect of higher education expansion on men’s level of education. At none of the levels of education is the joint hypothesis test significant at the 95% level. That higher education expansion increases girls’ educational opportunity at tertiary, high school, and middle school levels (as shown in “Appendix Table 7”) but has a limited effect on boys’ educational opportunity suggests that the chance of college admission might have presented a greater barrier to girls’ educational opportunities than boys. Previous research has shown that academic achievement matters for parents’ continuing investment in girls’ education but does not matter for boys’ persistence in the education system (Zhang et al., 2007). Starting from the compulsory-level education, academically weak boys stay in school longer than academically weak girls (Brown & Park, 2002). These findings suggest that girls’ educational opportunities might be particularly sensitive to the perceived likelihood of getting into college compared to boys, which helps explain why higher education expansion has a greater effect on women’s educational attainment.

To rule out the other potential threats to the exclusion restriction assumption, I conduct two falsification tests. First, as previously shown in “Appendix Table 7”, the higher education expansion only affected women’s attainment at the tertiary, high school, and middle school levels. It did not affect the probability of women attaining primary-level education. Therefore, for women with less than middle school education, their fertility outcomes should not have been affected by the higher education expansion, unless through factors other than their educational attainment. I estimate the effect of higher education expansion among the subsample of women with less than middle school education and show no effect on their number of children ever born (Column 2 of “Appendix Table 14”). The second falsification restricts the sample to women who had already left education before the expansion began. For those women, higher education expansion did not affect their educational attainment, and thus should not have affected their fertility, unless through other factors. Column 3 of “Appendix Table 14” showed no effect of higher education expansion on the number of children ever born among this subsample of women, lending further support to the exclusion restriction assumption that education only affects women’s fertility through increasing their years of education.

**Table 6:** Descriptive statistics: number of children ever born by the level of educational attainment

	Level of educational attainment			
	Primary or below	Middle school	High school	Tertiary or above
Mean number of children ever born	1.75	1.32	0.98	0.64
By category (%)				
0	3.46	6.29	19.36	41.65
1	35.35	58.82	64.71	52.75
2	48.22	31.71	14.78	5.18
3 or more	12.97	3.18	1.15	0.42
*N*	1041	1542	785	1198

**Table 7:** Effect of higher education expansion on women’s educational attainment at each level: coefficients on the interactions between cohorts and provincial rate of expansion from Probit models (base category is “Rate of expansion × control cohorts”)

	Educational attainment by level			
	(1)	(2)	(3)	(4)
	Tertiary	High school	Middle school	Primary school
Rate of expansion × cohorts				
Rate of expansion × partially treated	3.839†	3.876	3.642**	0.0791
	(2.165)	(2.561)	(1.294)	(3.430)
Rate of expansion × treated	6.791***	8.663***	11.11***	2.842
	(1.524)	(2.542)	(1.480)	(4.013)
*χ*^2^ statistic	23.95***	19.43***	56.51***	0.64
*N*	4566	4566	4460	4025

Standard errors are in parentheses and clustered by province

Control cohorts are aged 23–27, partially treated cohorts are aged 18–22, and treated cohorts are aged below 18 in 1998, just before the start of the expansion. All models control for 1-year birth cohort and province fixed effects, age at the time of the survey, non-agricultural hukou, interactions between cohorts and provincial level GDP in 1998, tertiary enrolment in 1998, and fertility policy intensity

The *χ*^2^-statistic tests the hypothesis that the coefficients on the interaction terms are jointly zero

^†^*p* < 0.10, ***p* < 0.01, ****p* < 0.001

**Table 8:** Estimated effect of education on women’s employment and hourly wages among employed

	Employed	
	(1)	(2)
	Probit	IV Probit
Years of education	0.0439***	0.0262
	(0.0103)	(0.101)
Wald test of exogeneity		0.06
*N*	4038	4038

	Hourly wage (log)	
	(1)	(2)
	OLS	2SLS
Years of education	0.136***	0.203***
	(0.00854)	(0.0512)
Wu-Hausman test of endogeneity		1.664
Overidentification test		1.142
*N*	2219	2219

Standard errors are in parentheses and clustered by province

All models control for 1-year birth cohort and province fixed effects, age at the time of the survey, non-agricultural hukou, interactions between cohorts and provincial level GDP in 1998, tertiary enrolment in 1998, and fertility policy intensity

****p* < 0.001

**Table 9:** Estimated effect of women’s education on non-wage income (log) and spouses’ education

	Non-wage income (log)	
	(1)	(2)
	OLS	2SLS
Years of education	0.0725***	0.128**
	(0.00727)	(0.0450)
Wu-Hausman test of endogeneity		0.675
Overidentification test		3.086†
*N*	3615	3615

	Spouse's years of education	
	(1)	(2)
	OLS	2SLS
Years of education	0.571***	0.581***
	(0.0153)	(0.115)
Wu-Hausman test of endogeneity		0.006
Overidentification test		0.947
*N*	3943	3943

Standard errors are in parentheses and clustered by province

All models control for one-year birth cohort and province fixed effects, age at the time of the survey, non-agricultural hukou, interactions between cohorts and provincial level GDP in 1998, tertiary enrolment in 1998, and fertility policy intensity

^†^*p* < 0.10, ***p* < 0.01, ****p* < 0.001

**Table 10:** Multinomial Logit Model estimates of the relationship between educational level and agreeing or disagreeing with the statement “The number of children one has is a personal matter. The government should not intervene” (base category is “neither agree nor disagree”). *Source*: CGSS (2010)

	“The number of children one has is a personal matter. The government should not intervene”	
	(1)	
	Agree	Disagree
Level of education (Primary or less)		
Middle school	0.183	0.0692
	(0.243)	(0.264)
High school	− 0.0281	− 0.103
	(0.270)	(0.296)
Tertiary and above	− 0.263	− 0.425†
	(0.233)	(0.258)
Constant	2.112***	1.128***
	(0.182)	(0.197)
*N*	1855	

Standard errors are in parentheses

^†^*p* < 0.10,**p* < 0.05, ***p* < 0.01, ****p* < 0.001

**Table 11:** Estimated effect of women’s education on relative education and income

	Have equal or more education than spouse	
	(1)	(2)
	Probit	IV Probit
Years of education	0.149***	0.180*
	(0.00843)	(0.0659)
Wald test of exogeneity		0.17
*N*	3943	3943

	Ratio of own income to spouse's income	
	(1)	(2)
	OLS	2SLS
Years of education	0.0216***	0.0725
	(0.00551)	(0.0516)
Wu-Hausman test of endogeneity		0.643
Overidentification test		0.177
*N*	3204	3204

Standard errors are in parentheses and clustered by province

All models control for 1-year birth cohort and province fixed effects, age at the time of the survey, non-agricultural hukou, interactions between cohorts and provincial level GDP in 1998, tertiary enrolment in 1998, and fertility policy intensity

**p* < 0.05, ****p* < 0.001

**Table 12:** Placebo tests: Effects of higher education expansion on older cohorts aged 23–32 in 1998 from OLS regression models (base category is “Rate of expansion × aged 28–32 in 1998”)

	Years of education		
	(1)	(2)	(3)
Rate of expansion × cohorts			
Rate of expansion × aged 23–27 in 1998	− 0.435	− 3.087	− 3.418
	(3.047)	(2.913)	(3.326)
Controls			
Age at the time of the survey	No	Yes	Yes
Non-agricultural hukou	No	Yes	Yes
Han majority	No	Yes	Yes
Interaction between exposure and 1998 GDP	No	No	Yes
Interaction between exposure and 1998 enrolment	No	No	Yes
Interaction between exposure and fertility policy intensity	No	No	Yes
*N*	3418	3418	3418

All models control for one-year birth cohort and province fixed effects, age at the time of the survey, non-agricultural hukou, interactions between cohorts and provincial level GDP in 1998, tertiary enrolment in 1998, and fertility policy intensity. Standard errors are in parentheses and clustered by province

**Table 13:** Effect of higher education expansion on men’s educational attainment at each level: coefficients on the interactions between cohorts and provincial rate of expansion from Probit models (base category is “Rate of expansion × control”)

	Education attainment of males by level			
	(1)	(2)	(3)	(4)
	Tertiary	High school	Middle school	Primary school
Rate of expansion × cohorts				
Rate of expansion × partially treated	0.888	1.408	1.367	2.290
	(1.973)	(1.933)	(2.485)	(4.709)
Rate of expansion × treated	4.412*	3.731†	4.859*	8.242
	(2.059)	(2.074)	(2.467)	(6.103)
*χ*^2^ statistic	5.21†	3.58	3.13	1.09
*N*	3973	3973	3864	2138

Standard errors are in parentheses

Control cohorts are aged 23–27, partially treated cohorts are aged 18–22, and treated cohorts are aged below 18 in 1998, just before the start of the expansion. All models control for 1-year birth cohort and province fixed effects, age at the time of the survey, non-agricultural hukou, interactions between cohorts and provincial level GDP in 1998, tertiary enrolment in 1998, and fertility policy intensity

The *χ*^2^-statistic tests the hypothesis that the coefficients on the interaction terms are jointly zero

^†^*p* < 0.10, **p* < 0.05

**Table 14:** Falsification tests: effect of higher education expansion on number of children ever born (base category is “Rate of expansion × control”). Coefficients on the interactions between cohorts and provincial rate of expansion from Poisson models estimated among (1) full sample of somen (2) subsample of women with primary education and below and (3) subsample of women who had left school before 1999

	Children ever born		
	(1)	(2)	(3)
	Full sample	women with primary education and below	women who had left school before 1999
Rate of expansion × cohorts			
Rate of expansion × partially treated	0.522	0.676	0.351
	(0.711)	(1.097)	(0.601)
Rate of expansion × treated	2.588***	− 2.406†	− 0.138
	(0.719)	(1.337)	(0.944)
*χ*^2^ statistic	13.68**	4.67†	0.42
*N*	4566	1041	2145

Control cohorts are aged 23–27, partially treated cohorts are aged 18–22, and treated cohorts are aged below 18 in 1998, just before the start of the expansion. All models control for one-year birth cohort and province fixed effects

Standard errors are in parentheses and clustered by provinces. †*p* < 0.10 ***p* < 0.01 ****p* < 0.001. The *χ*^2^ statistic tests the hypothesis that the coefficients on the interaction terms are jointly zero

## Footnotes

1 By the end of the 1990s, only about a third of the population was estimated to be subject to a strict one-child policy (Gu et al., 2007). By 2007, in all provinces but one, couples who were both only-children themselves are permitted to have two children (Zeng & Hesketh, 2016).

2 Marriage is near-universal in China (Jones, 2007; Yeung & Hu, 2016), and non-marital childbearing is extremely rare.

3 Empirical evidence of a “quantity-quality tradeoff” is mixed (Angrist et al., 2010; Black et al., 2005; Kugler & Kumar, 2017; Li et al., 2008; Ponczek & Souza, 2012; Rosenzweig & Wolpin, 1980; Rosenzweig & Zhang, 2009).

4 The conclusions hold even after taking into account the child quantity-quality interaction (Ermisch, 1989; Hazan & Zoabi, 2015).

5 Without the lead-in “In the absence of the one-child policy…”, a conventional measure of fertility intention underestimates the “true” demand for children in the Chinese context, since respondents may simply restate government restrictions on family size or provide socially desirable answers (Feng, 2017; Hermalin & Liu 1990; Merli & Smith, 2002; Morgan et al., 2009).

6 Reforms to the *hukou* systems have made possible changes of *hukou* location (or place of registration); however, data from 2000 census suggest that the total number of individuals who have changed their *hukou* from one province to another, i.e. permanent interprovincial migrants, account for only 0.36% of the total population (Fan, 2005; Sun & Fan, 2011).

7 Although a number of public universities directly under the administration of the Chinese central government recruit students from across the country, the main driver of the higher education expansion is public universities under the jurisdiction of the provincial governments and private institutions, which admit majority of the students from within the province (Carnoy et al., 2013, 49).
